# Investigation of cyclic water infiltration and dry-out in coated spruce using finite-element simulations

**DOI:** 10.1007/s00226-025-01629-7

**Published:** 2025-01-22

**Authors:** Florian Brandstätter, Magdalena Senoner, Markus Lukacevic, Maximilian Autengruber, Michael Truskaller, Gerhard Grüll, Josef Füssl

**Affiliations:** 1 TU Wien, Institute for Mechanics of Materials and Structures, Karlsplatz 13, Vienna, 1040 Austria; 2 Holzforschung Austria, Franz Grill-Strasse 7, Vienna, 1030 Austria

## Abstract

Accurate prediction of moisture distributions in wood is among the most critical challenges in timber engineering. Achieving this requires a well-coordinated comparison of experimental methods and simulation tools. While significant progress has been made in developing simulation tools in recent years, a lack of experience with and trust in these tools continues to hinder broader implementation, especially when it comes to free water and its absorption. Investigations and model advancements have allowed for the simulation of increasingly complex cases, including one-dimensional moisture transport above the fiber saturation point (FSP) in coated boards and below FSP in coated glued laminated timber (GLT). However, free water flow in coated GLT beams has not yet been addressed, which can become problematic in case of extreme scenarios, such as water infiltration. In this study, we demonstrate that the multi-Fickian free water transport model developed by some of the authors can successfully simulate three-dimensional coated cases. Uncoated and coated boards and GLT members were subjected to cyclic wetting and drying, both experimentally and numerically. To simplify the calibration process of the mass transfer coefficient of free water—identified as the most significant parameter for the simulation of free water transport—experiments previously conducted by some of the authors were simulated. Based on the simulation results, approaches for an initial estimation of the mass transfer coefficient were developed. If the water uptake of coated specimens is measured three days after continuous soaking in water and the result exceeds a specific limit, the coefficient can be sufficiently predicted. The simulation and experimental results show a good agreement.

**Supplementary Information:**

The online version contains supplementary material available at 10.1007/s00226-025-01629-7.

## Introduction

Wood has been a fundamental building material for centuries due to its availability, strength, and high strength-to-weight ratio (Asdrubali et al. 2017). In modern construction, engineered wood products, such as glued laminated timber (GLT), have emerged as innovative solutions to create large-span and high-rise structures. However, wood’s natural hygroscopicity—its tendency to absorb and release moisture—remains a challenge in timber engineering, as fluctuations in moisture content (MC) can lead to swelling and shrinkage, resulting in internal stresses due to wood’s orthotropic material behavior. GLT beams, commonly used as load-bearing components in timber structures, are potentially subjected to significant climatic loads. While leakages and burst water pipes can considerably increase MC, dry winter climates may reduce it to levels where moisture-induced deformations cause cracking (Dietsch et al. 2015). In the worst case, structural failure occurs.

In response to these challenges, protective coatings can be applied to GLT beams to reduce moisture ingress and, therefore, moisture-induced stresses (Fragiacomo et al. 2011). These coatings act as barriers, mitigating the impact of moisture and enhancing the durability of wood in exposed conditions. However, several factors affect the performance of coatings. Not only does the type of coating affect water infiltration (Ahola et al. 1999; de Meijer and Militz 2001; Ekstedt 2003; Grüll et al. 2013; Gezici-Koç et al. 2019; Sjökvist and Blom 2019; Sjökvist et al. 2019a, b, 2020), but also combinations in coating systems (Angelski 2019). In addition, increasing the number of coating layers rather than the total thickness of the coating decreases the amount of water uptake (Hýsek et al. 2018; Angelski 2019; Grüll et al. 2010; Senoner 2024). Depending on the coating, there can be differences. For some coatings, a third layer affected water uptake only minimally compared to two applied layers (Angelski 2019; Grüll et al. 2010; Senoner 2024), while a significant influence on water infiltration by a third layer can be seen for others (Angelski 2019). Furthermore, the coating system (Ekstedt 2003; Grüll et al. 2013; Miklečić and Jirouš-Rajković 2021), the pigmentation level (Huldén and Hansen 1985; Ekstedt 2003), the spreading rate (Senoner 2024), the substrate (De Windt et al. 2009) and the viscosity of the coating (Shin and Lee 2020) change moisture uptake.

The variability in moisture ingress of coatings underscores the careful consideration of its potential performance level. Disproportional high moisture absorption resistance values to protect GLT beams can become problematic when drying out is impeded and, thus, moisture trapping conditions are created (de Meijer 2002). Therefore, water vapor desorption should be allowed to a certain degree to prevent moisture retention, while minimizing moisture-induced deformations and, thus, cracking. Evaluating an optimum for water absorption and desorption to prevent water ingress and moisture-induced stresses requires significant effort. Above all, it demands tools capable of quickly and easily calculating moisture distributions in wood, enabling the efficient evaluation of various boundary conditions. Physical experiments are well-suited for representing realistic conditions but are both time-consuming and costly. They do not allow for extensive parameter studies and can only capture moisture distributions in materials selectively.

In contrast, numerical simulations offer almost limitless possibilities. However, their widespread adoption is hindered by a lack of experience regarding the performance of these methods. The complexity of input parameters and the intricate transport and transition conditions—particularly when accounting for free water—have so far limited the support simulation programs can provide to research and development tasks.

Only through complementary studies that compare experimental observations with simulation results, critically assess input parameters, and highlight the strengths and weaknesses of these methods can sufficient trust be built to establish integrative concepts for the future. Given the critical role of moisture in timber engineering, advancing these developments cannot be overstated. This work aims to contribute to this effort.

Several studies have employed numerical models to investigate moisture transport in wood (e.g.: Angst and Malo 2013; Florisson et al. 2020; Fortino et al. 2013; Konopka and Kaliske 2018; Niklewski et al. 2016; Niklewski and Fredriksson 2021) but only a few focused on predicting moisture distributions in coated test specimens using numerical methods. Virta et al. (2006) examined the change in MC of wooden cladding boards during short-term water soaking using a single-Fickian numerical approach. They assumed one-dimensional moisture transport in transverse direction, where water flow was solely based on Darcy’s law, neglecting effects caused by diffusion and air pressure. Experimental results were used to calibrate the apparent surface emission coefficient to account for the influence of the omitted air pressure. Overall, the study showed a good agreement between experimental and simulation results and suggested a coupled capillary diffusion approach for analyzing moisture distribution in wood. Fortino et al. (2019) extended a numerical model to analyze the MC development in coated wooden bridge members (GLT beams), which were exposed to outdoor climate conditions but protected from rain. Thus, it was assumed that no free water transport occurred. The studies showed advances in the numerical simulation of moisture transport in coated timber elements. However, the moisture development above the fiber saturation point (FSP) in coated GLT beams subjected to intense wetting and drying cycles to examine potential crack development caused by moisture-induced stresses remains to be investigated.

Thus, this study aims to combine practical experiments with numerical simulations to analyze moisture fluctuations in coated GLT beams subjected to cyclic water infiltration (above FSP) and subsequent drying, as well as the resulting crack development. For the MC simulations, the free water transport model of Autengruber et al. (2020) is used, which has not yet been applied to coated timber elements. Simulating free water transport in case of infiltration using this model requires a time-consuming calibration of the mass transfer coefficient of free water k$_{\mathrm {c_w}}$. It considers infiltration resistances of free water flux by, e.g., coatings, and, therefore, significantly influences water uptake, as identified in Brandstätter et al. (2023). Previous studies revealed a wide range of k$_{\mathrm {c_w}}$ values (Autengruber et al. 2020; Brandstätter et al. 2023). To reduce the effort determining k$_{\mathrm {c_w}}$, one objective of the study is to examine and simulate the experiments of Grüll et al. (2010, 2015), which investigated the water uptake in coated timber boards. Based on these simulations, suggestions are presented to estimate k$_{\mathrm {c_w}}$, which are used to calibrate k$_{\mathrm {c_w}}$ for the experiments conducted in this study. Subsequently, the experimental and simulation results are compared.

To simulate fracture, stress simulations, including surface-based cohesive behavior and XFEM (eXtended Finite Element Method) to allow for discrete cracking, are performed, which use the results of the MC simulations as loads. The failure of wood is defined by a multisurface failure criterion from Lukacevic et al. (2015, 2017); Lukacevic and Füssl (2016) and Li et al. (2018), and moisture-dependant stiffness parameters are determined with the multiscale material model of Hofstetter et al. (2005).

## Materials and methods

In the following, the experiments, as well as the models to simulate moisture transport and fracture in wood are introduced. In addition, the investigated timber members’ geometries, the corresponding finite element model, and the hygrothermal loads are presented.

### Experiments

First, the most essential information from the already conducted experiments of Grüll et al. (2010, 2015) is introduced for the estimation of k$_{\mathrm {c_w}}$. Then, the experiments to evaluate the required performance of the coatings are presented (pretests), followed by the ones investigating the water uptake and dry-out in coated GLT members (main tests).

#### Collection of existing coating data

In the technical report of the Woodexter project - Work Package 3 (Grüll et al. 2010), the effects of coatings on wood product performance were studied. Among the studied issues were liquid water (according to ÖNORM EN 927-5 (2007)) and water vapor permeability tests (according to EN ISO 12572 (2016)). For the spruce samples, an average dry density of 490 kg m$^{-3}$ was determined. For the liquid water permeability tests, the boards ($95 \times 22$ mm) were preconditioned at 20 $^{\circ }$C and 65 % RH until the mass was constant. Subsequently, the boards were stored in water for 72 h, followed by 120 h in a climate chamber at 20 $^{\circ }$C and 65 % RH. For the water vapor permeability tests, the dry-cup-method was used, where the boards were preconditioned at 23 $^{\circ }$C and 50 % RH. In Table 1, the $s_\textrm{d}$ values for all investigated coating configurations (water-based acrylic stains and paints) are shown. In addition, the water uptakes after three days $\Delta \,m_{\textrm{3d}}$ for each case are presented, where for the uncoated panels an average uptake of 998 g m$^{-2}$ was determined.

**Table 1:** $s_\textrm{d}$ values and water uptake after three days $\Delta \,m_{\textrm{3d}}$ for each configuration of the Woodexter experiments.

Coatings	P20	P50	P80	R50	W50	W100
$s_\textrm{d}$ values (m)	0.24	0.69	0.85	0.86	0.89	1.62
$\Delta \,m_{\textrm{3d}}$ (g m$^{-2}$)	543	481	496	444	356	340

The numbers define the target thickness of the coating layers in $\upmu \hbox {m}$, while the single letters specify the colors of waterborne acrylic dispersions: P = brown, R = red and W = white

All coatings were waterborne acrylic dispersions, which were applied with an airless spray gun in one (P20) or two layers (P50, P80, W50, W100, R50). The letter indicates the color of the applied waterborne acrylic dispersion (P = brown, R = red, W = white), while the number defines the target thickness of the coating layers in $\upmu \hbox {m}$.

Another technical report of Grüll et al. (2015) compares historical and modern coating systems in terms of ecologic and technical properties. As described above, the liquid water permeability tests according to ÖNORM EN 927-5 (2007) and water vapor tests according to EN ISO 12572 (2016) were performed. For the liquid water tests, the following seven different coatings were investigated: boiled linseed oil (BLO), linseed oil-stand oil (LOSO), opaque solvent-based alkyd (OAk), opaque acrylic-/alkyd hybrid (OAcAk), transparent solvent-based alkyd (TAk), transparent acrylic (TAc) and transparent acrylic-/alkyd hybrid (TAcAk). The corresponding $s_\textrm{d}$ values determined in the water vapor tests and the calculated $\Delta \,m_{\textrm{3d}}$ of each configuration can be obtained from Table 2, where for the uncoated boards an average uptake of 765 g m$^{-2}$ was measured. The size of the panel and the dry density were not given, which is why a cross-sectional size of 7 $\times$ 2 cm, as proposed in ÖNORM EN 927-5 (2007), and a dry density of 420 kg m$^{-3}$ were assumed. In this work, these experiments will be referred to as CHMC (Comparison of Historic and Modern Coatings).

**Table 2:** $s_\textrm{d}$ values and water uptake after three days $\Delta \,m_{\textrm{3d}}$ for each configuration of the coatings from Grüll et al. (2015)

Coatings	BLO	TAc	TAcAk	TAk	LOSO	OAcAk	OAk
$s_\textrm{d}$ values (m)	0.7	0.9	1.3	2.5	3.4	3.4	6.9
$\Delta \,m_{\textrm{3d}}$ (g m$^{-2}$)	356	43	33	79	61	147	132

BLO = boiled linseed oil, LOSO = linseed oil-stand oil, OAk = opaque solvent-based alkyd, OAcAk = opaque acrylic-/alkyd hybrid, TAk = transparent solvent-based alkyd, TAc = transparent acrylic, TAcAk = transparent acrylic-/alkyd hybrid

#### Pretests

**Fig. 1:**
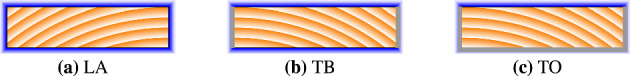
Overview of the coating configurations of the board test specimens. **a** The lateral surface area is defined as water absorption surface; **b** The top and bottom surfaces are the water absorption surfaces. The side surfaces are sealed; **c** The top surface is the only water absorption surface, the remaining ones are sealed

For the pretests, three coating configurations with different water absorption surfaces and two coating materials—a solvent-based alkyd stain (SAk) and a water-based acrylic paint (WAc)—were examined. Figure 1 shows the studied coating configurations. For the LA configuration, the lateral surface area defined the water absorption surfaces (Fig. 1a). For TB, the top and bottom surfaces were the water absorption surfaces (see Fig. 1b), while only the top surface was the water absorption surface for the configuration TO (Fig. 1c).

For all configurations, the end-grain surfaces, as well as the surfaces not mentioned in the configuration name, were sealed with three layers of a grey 2K epoxy sealer. To identify the difference a coating causes in moisture uptake, the boards were divided into two equal-sized test specimens with mirrored fiber orientation pattern, as indicated by Fig. 1a and b. For one, the water absorption surfaces were coated, while for the other, the water absorption surfaces were left untreated (matched samples). The resulting 36 test specimens were made of Norway spruce wood (Picea abies L.), with a width of 80 mm, a height of 20 mm, and a length of 170 mm. The growth ring orientation was between 5 $^{\circ }$ and 45 $^{\circ }$ to the top surface and the (theoretical) pith was about 200 mm distant from the considered section, with the bottom surface facing the pith (see Fig. 1).

**Fig. 2:**
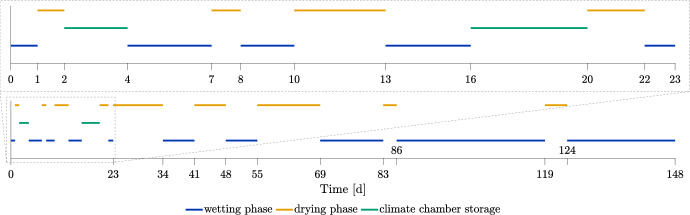
Overview of the pretest’s wetting and drying cycle durations

All test specimens were subjected to various wetting and drying cycles, where for each wetting phase, the test specimens were submerged completely in water, whereas for each drying phase, the test specimens were put into a drying oven (Heraeus UT6120). The temperature of the drying oven was set to 50 $^{\circ }$C. However, for the last two drying cycles (days 83 to 86 and 119 to 124), the temperature was increased to 70 $^{\circ }$C. The duration of each phase can be obtained from Fig. 2. Twice, the test specimens were stored in a climate chamber with a relative humidity (RH) of 65 % and a temperature of 20 $^{\circ }$C. After each phase, the test specimens were weighted with a Sartorius GP5202.

After the experiments, tests according to DIN EN ISO 7783 (2019) were performed to determine the $s_\textrm{d}$ values of the dispersions, resulting in 0.25 m for the SAk, and 0.9 m for the WAc coating material.

#### Main tests

**Fig. 3:**
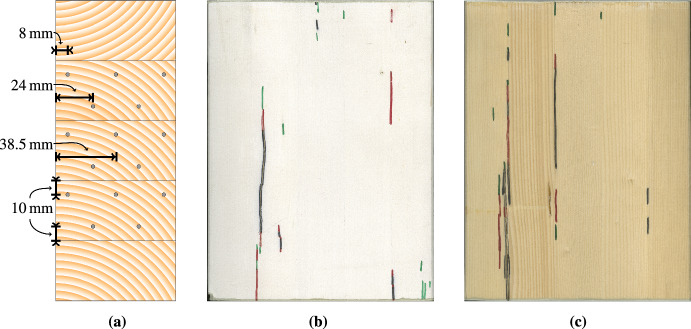
**a** Schematic cross section of the GLT samples, including the locations of the measuring electrodes illustrated with grey dots. **b** Crack formation after the third drying phase for a coated test specimen, and **c** the corresponding uncoated matched sample

For the main tests, samples from GLT beams were subjected to three wetting and drying cycles each to investigate moisture-induced crack development with and without laterally coated surfaces. To examine the coating influence on moisture changes, matched samples were manufactured for this experiment. The original GLT beams with an approximate width of 160 mm, a height of 200 mm and a length of about 3.50 m were cut in half along a vertical line in the middle of the cross section. From the resulting pairs of beams equal in size, matched samples with a length of about 250 mm were produced, resulting in a total of 18 GLT test specimens. To track swelling and shrinkage, the specimens were scanned weekly. The height and width of the specimen’s cross section were measured using the ImageJ software Fiji (Schindelin et al. 2012). The results reveal an average initial width of 78 mm and an average initial height of 198 mm for all test specimens. At the end of each drying phase, the crack development was documented the same way. The GLT test specimens were made of Norway spruce wood (Picea abies L.) with five lamellas. The piths were located in the middle of the bottom edges of the original lamellas, with a flipped one at the top. When producing the test specimens, the pith was removed (see Fig. 3a).

For this series of tests, an opaque white acrylic water-based experimental formulation (OWAc) was examined, with a varying number of coating layers from 0 to 3 (each with a spreading rate of 75 g m$^{-2}$), and, therefore, differences in the target coating thickness (see Table 3 for details). To exclude unwanted water uptake, the end-grain surfaces of each specimen were sealed with three layers of a 2K epoxy sealer.

**Table 3:** Characteristics for the coating configurations of the main tests (OWAc = opaque white acrylic water-based experimental formulation). The number defines the target thickness of the coating configuration ($\upmu \hbox {m}$)

Coating configuration	Untreated	OWAc20	OWAc40	OWAc60
Number of layers	0	1	2	3
Total spreading rate (g m$^{-2}$)	–	75	150	225
Target coating thickness ($\upmu \hbox {m}$)	–	20	40	60
$s_\textrm{d}$ value (m)	–	0.02	0.08	0.14

In a cyclic test, the GLT members were subjected to a wetting phase followed by a drying phase. This cycle was repeated three times, where each phase lasted seven days (42 days in total). In the wetting phase, the GLT members were immersed in a water bath, and, in the drying phase, put in a drying oven (Heraeus UT6120) at 80 $^{\circ }$C. Each weekday (Monday to Friday), the masses of the GLT members were weighted with a Sartorius GP5202, and the wood moisture content was manually measured at 15 different positions (see Fig. 3a) with a Brookhuis FME moisture meter. The measurements were taken at three different depths from the surface (8 mm, 24 mm and 38.5 mm) of each side surface, with a vertical distance from the corresponding glue lines of 10 mm. For the measurements, custom-made electrodes were produced (for details, see Senoner 2024).

In addition, at the end of each drying phase, the cracks on the side surfaces initiated during this period were highlighted with pens in different colors to identify the point in time of crack development (see Fig. 3b and c). Black was used for the first cycle, red for the second, and green for the third one. It can be seen that most of the wood cracks initiated along the glue lines. Following measurements revealed that delamination of the glue lines can be excluded and, thus, solely wood failed (Senoner 2024).

### Mathematical model for moisture transport in wood

Based on the work of (Eitelberger et al. 2011; Fortino et al. 2013, 2019; Frandsen et al. 2007a; Konopka and Kaliske 2018; Krabbenhøft and Damkilde 2004b), Autengruber et al. (2020) developed a multi-Fickian moisture transport model including free water flow, which was used for the simulations. The change in MC is described by the following three phases: bound water, water vapor and free water. While the transport of bound water and water vapor is based on the multi-Fickian theory (diffusion processes below FSP), free water flow (above FSP) is considered with a mixed formulation, combining a pressure-driven (Darcy’s law) and concentration-based (diffusion) process. The phases are coupled via condensation, evaporation and sorption rates, including the consideration of hysteresis effects, and are assumed continuous within a representative volume element. To account for the impacts of phase changes and thermal conduction, an energy conservation equation was implemented, resulting in four coupled differential equations. These were implemented in the commercial finite element software Abaqus (Abaqus Documentation 2014) using the user-element subroutine UEL and were solved with the modified Newton method.

To determine the equilibrium moisture content EMC and, thus, estimate the RH in the oven used in the experiments, the following sorption isotherm equation of Hailwood and Horrobin (1946) (see Eq. (1)) and the temperature-dependent adsorption isotherm from Zuritz et al. (1979) (see Eq. (2)) were used:

1 $$\begin{aligned} \textrm{EMC}_{s}= & \frac{\textrm{RH}}{f_{1,s} + f_{2,s} \, \textrm{RH} + f_{3,s} \, \textrm{RH}^2}. \end{aligned}$$

2 $$\begin{aligned} \textrm{EMC}&= 0.01\! \left( \frac{-\textrm{T}~\textrm{ln}(1-\textrm{RH})}{0.13 \! \left( 1- \left( \frac{\textrm{T}}{647.1}\right) ^{-6.46}\right) }\right) ^{1/(110\,\textrm{T}^{-0.75})} \end{aligned}$$

with shape factors experimentally determined by Frandsen et al. (2007b). For adsorption ($s = a$), the values are $f_{1,a}=1.804, f_{2,a}=13.63$ and $f_{3,a}=-12.12$, and for desorption ($s = d$), $f_{1,d}=1,886, f_{2,d}=7.884$ and $f_{3,d}=-6.526$. T defines the temperature.

#### Initial conditions

Based on the initial temperature $\textrm{T}_{ini}$ and the initial relative humidity $\textrm{RH}_{ini}$, the initial water vapor concentration $c_{v,ini}$ was derived. Initially, an equilibrium between $c_{v,ini}$ and the concentration of bound water $c_{b,ini}$ was assumed, and, thus, $c_{b,ini}$ could be determined with the dry density $\rho _{\textrm{d}}$ under consideration of the adsorption isotherm (see Eq. (1)).

#### Boundary conditions

The influence of the surrounding climate on wood’s moisture development was considered with boundary conditions, which define the fluxes through the exchange surfaces. The fluxes of free water, water vapor and temperature were considered, with the free water flux $\phi _w$ given as:

3 $$\begin{aligned} \begin{aligned} \phi _w = k_{{c}_w} (c_w - c_{w,0}&)f_{lum} \end{aligned} \end{aligned}$$

where $k_{{c}_w}$ is the free water mass transfer coefficient, which considers possible resistances due to coatings, etc. $f_{lum}$ defines the volume proportion, which is filled with gas and water. $c_w$ is the free water concentration in the cell and $c_{w,0}$ the one of the surrounding fluid.

The water vapor flux $\phi _v$ is described as

4 $$\begin{aligned} \begin{aligned}&\phi _v = k_{{c}_v} (c_v - c_{v,0})f_{lum} \\&k_{{c}_v} = \frac{1}{\frac{1}{k_{{c}_v,surf}} + \frac{1}{k_{{c}_v,coat}}} \\&k_{{c}_v,surf} = Sh\,D_{air}/L \\&k_{{c}_v,coat} = D_{air}/s_\textrm{d} \end{aligned} \end{aligned}$$

with the water vapor concentration of the surrounding climate $c_{v,0}$ and the film boundary coefficient $k_{{c}_v}$. It considers convection and airflow depending on airspeed ($k_{{c}_v,surf}$), and the influence of potential coatings ($k_{{c}_v,coat}$). $k_{{c}_v}$ was determined according to the formulation of Autengruber et al. (2021), which was based on Fortino et al. (2019) and Häglund (2010). $k_{{c}_v,surf}$ was obtained from Eitelberger et al. (2011), with the dimensionless Sherwood number *Sh* set to 1 (Bird et al. 2002), the diffusion coefficient of water vapor in air $D_{air}$ (m$^2$ s$^{-1}$) as defined in Eq. 2 in Online Resource 1 (Schirmer 1938), and a characteristic length *L* (Bird et al. 2002). *L* corresponds to the distance between a liquid releasing water vapor and a piece of wood for moisture uptake tests, which was 0.035 m (Dvinskikh et al. 2011). For the cup tests of the Woodexter experiments, the test specimens were subjected to an intense air flow of 0.12 m s$^{-1}$ in a climate chamber and, therefore, L was decreased to 0.012 m for the simulations. $k_{{c}_v,coat}$ was taken from Autengruber et al. (2021), with $s_\textrm{d}$ defining the water vapor diffusion-equivalent air layer thickness (m) (EN ISO 12572 2016), which was calculated based on cup tests according to DIN EN ISO 7783 (2019). The $s_\textrm{d}$ value changes with each new coating configuration and, thus, needs to be known for the respective simulations.

In addition to the free water and water vapor flux, the flux of energy was also considered (see Autengruber et al. (2020) for more details).

#### Glue lines

For the glue lines of the GLT members examined in the main tests, melamine urea formaldehyde (MUF) was used, which influences diffusion and capillary moisture transport processes only minimally (Volkmer et al. 2012; Mannes et al. 2012). Therefore, it was assumed that the diffusion and permeability parameters are not affected by the adhesives.

### Mathematical model for fracture in wood

For the determination of fracture, a subsequent stress simulation based on linear elastic material behavior was performed, where the moisture simulations were used as a loading. Based on the moisture-induced strains, stresses were determined in each integration point of each increment. As dimensional changes primarily occur in the hygroscopic range (Neuhaus 2017), only variations in $c_b$ induced stresses, which can cause crack development.

To simulate discrete cracking, two node separation methods were used: the extended finite element method (XFEM) and surface-based cohesive behavior. Within the framework of XFEM, additional degrees of freedom and specific enrichment functions allow to consider discontinuities and, thus, cracking. In Abaqus, all elements that are supposed to crack have to be assigned to a so-called enrichment region. The cohesive surfaces are implemented between the lamellas and the glue lines, considering linear elastic traction-separation behavior.

As soon as the stresses, determined for each increment, exceed the corresponding strength criterion, node separation is initiated. For XFEM, the strength of wood was considered by a multisurface failure criterion from Lukacevic et al. (2017). Crack propagation was allowed and multicracks were activated with a specific crack initiation radius. For the cohesive surfaces, a maximum stress criterion was used with a normal strength of 5 N mm$^{-2}$ and a shear strength of 6 N mm$^{-2}$. An energy-based damage evolution was defined with a fracture energy of 0.6 N mm mm$^{-2}$ and all stiffness components were set to 10,000 N mm$^{-3}$. For the damage stabilization, a viscosity coefficient of 1$\cdot \,10^{-5}$ was assumed.

Due to the intense moisture changes, significant moisture-induced stresses causing crack development were expected. In addition, the presented experiments suggest long computation times, which should ideally be avoided. Therefore, for each stress simulation used to investigate fracture, an additional one was performed to determine the so-called crack-prone volume. The crack-prone volume is the sum of all elements that violate the multisurface failure criterion per increment. Thus, the higher the crack-prone volume, the deeper cracks can be assumed. Based on the crack-prone-volume data, the points in time when the deepest cracks can be expected were evaluated. At these points, the fracture simulations were performed.

In each integration point, a cylindrical-orthotropic coordinate system was defined, considering the material orientation of the corresponding test specimen.

Initially, stress-free cross sections were assumed due to uniform moisture conditions.

#### Material properties

To consider the moisture-dependent material properties of wood, the elastic material tensors of Hofstetter et al. (2005) for Norway spruce (Picea abies) with a clear wood dry density of 420 $\mathrm {kg\,m^{-3}}$ were used for the simulations. The tensor components were determined based on a continuum micromechanics model for MC levels ranging from 3 % to 30 % in 1 % increments (see Table S5 in Online Resource 1).

#### Multisurface failure criterion

For XFEM, the strength of wood is defined by the multisurface failure criterion from Lukacevic et al. (2015, 2017); Lukacevic and Füssl (2016) and Li et al. (2018), which was developed at the annual ring scale considering the structural features of late- and earlywood. The multisurface failure criterion is based on the work of Tsai and Wu (1971), whose definition of the failure surfaces was further developed to:

5 $$\begin{aligned} f_\textrm{i}^{\textrm{cw}} \left( \mathbf {\sigma } \right)&= a_{\textrm{LL,i}} \, \sigma _{\textrm{LL}} + a_{\textrm{RR,i}} \, \sigma _{\textrm{RR}} \\ &\quad + a_{\textrm{TT,i}} \, \sigma _{\textrm{TT}} + b_{\textrm{LLLL,i}} \, \sigma ^2_{\textrm{LL}} + b_{\textrm{RRRR,i}} \, \sigma ^2_{\textrm{RR}} \\ &\quad + b_{\textrm{TTTT,i}} \, \sigma ^2_{\textrm{TT}} + 2b_{\textrm{RRTT,i}} \, \sigma _{\textrm{RR}} \, \sigma _{\textrm{TT}} + 4b_{\textrm{LRLR,i}} \, \tau ^2_{\textrm{LR}} \\ &\quad + 4b_{\textrm{RTRT,i}} \, \tau ^2_{\textrm{RT}} + 4b_{\textrm{TLTL,i}} \, \tau ^2_{\textrm{TL}} \le 1 \end{aligned}$$

**Fig. 4:**
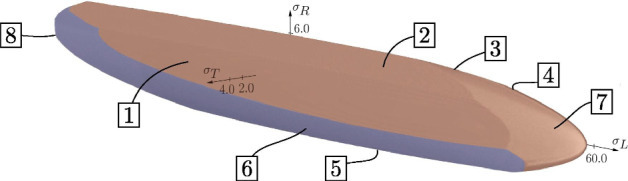
3D representation of the failure surfaces from Lukacevic et al. (2017). Stresses are displayed in the $\sigma _{\textrm{L}}-\sigma _{\textrm{R}}-\sigma _{\textrm{T}}$ stress space

With this criterion, brittle (cracking) as well as ductile (plastic) failure behavior can be considered. For brittle failure, the normal vector of the corresponding failure surface (1, 2, 3 and 7) defines the orientation of the cracks (see Fig. 4). The ellipsoidal shape of the failure surfaces is based on the direction-dependent strength of wood. In longitudinal direction, the strength is about 56 MPa, whereas in radial direction, the strength is approximately 5 MPa. In tangential direction, the strength is about 2 MPa. The components of the failure criterion were developed based on an MC of 12%, and do not change with the MC. As in case of cracking the MC in drier zones is approximately 12%, the error of not adapting the failure surface to the MC level is negliable small.

### Geometries and finite element models

**Fig. 5:**
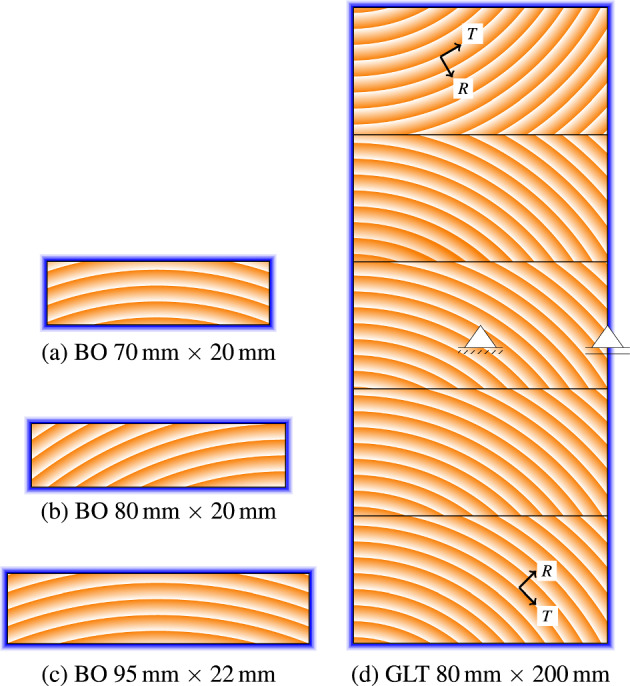
Illustration of the geometric boundary conditions, the locations of piths as well as the definition of local coordinate systems of the studied cross sections. The blue frames show where the boundary conditions are applied. **a** CHMC, **b** pretests, **c** Woodexter and **d** main tests

The simulations were performed on three different boards and one glued laminated timber cross section, which dimensions are summarized in Table 4. The locations of the piths vary with the experiments (see Fig. 5). While the (theoretical) pith for the boards of the Woodexter and CHMC experiments is approximately 200 mm below the middle of the bottom edge, the pith of the pretest’s boards is additionally shifted horizontally to the corresponding corner. For the GLT cross section, the piths of the lamellas are distributed along a vertical axis that is 2 mm from the left edge of the cross section, where the perpendicular distance of each lamella’s pith is about 50 mm from the top edge. The GLT cross section consists of five lamellas, each 40 mm high, with the top lamella flipped, as shown in Fig. 5.

The geometric boundary conditions are defined by a hinged support in the center point and a roller support in the middle of the right edge of the cross section (see Fig. 5). As plane strain conditions are set, no deformations out-of-plane occur. It is assumed that initially no eigenstresses from, e.g., production processes are present.

Due to the different dimensions, the finite element models vary. For all boards, the height and width of the elements is 1 mm, while for the GLT cross section, height and width range from 2 mm to about 4 mm to obtain an optimum in accuracy and computation time. Close to the edges, the element dimensions are minimal, increasing towards the center. The total number of elements can be obtained from Table 4.

For all simulations, the type of all elements is an eight-node brick with linear shape functions. In longitudinal direction, only one element with a depth of 1 mm is given to model plane strain conditions.

**Table 4:** Dimensions and mesh data of the cross sections

	Pretests	Maintests	Woodexter	CHMC
Width (mm)	80	80	95	70
Height (mm)	20	200	22	20
Number of finite elements	1600	1674	2090	1400

### Hygrothermal load

To consider the given climatic conditions of the experiments, RH and temperature data were applied as boundary conditions, and the mass transfer coefficient of free water $k_{{c}_w}$ was calibrated to simulate water uptake. For the Woodexter and the CHMC experiments, the boundary conditions were available, while for the pre- and main tests, only the temperature was recorded. The oven’s temperature could be manually regulated, but the RH remained unknown. Therefore, it was determined based on the measured MC at the end of the main tests’ drying phases using the desorption isotherm (see Eq. (1)). The resulting boundary condition values of the main tests are summarized in Table 5. For the pretests, the RH levels were estimated based on the introduced temperature-dependent isotherm (see Eq. (2)) and the RH level of the main tests at the end of the last drying phase. The percentage increase of the resulting MC with temperature (323 K and 343 K) was used to determine the corresponding RH level at an RH of 15 % at 353 K (80 $^{\circ }$C). Thus, the RH levels were set to 20 % RH at a temperature of 323 K (50 $^{\circ }$C), and to 17 % at a temperature of 343 K (70 $^{\circ }$C). Measurements of former experiments revealed non-linear decreasing RH and increasing temperature levels once the drying phases had begun. Therefore, temperature and RH were approximated by linearly reducing the values in a stepwise manner for both pre- and main tests. The steps of RH and temperature levels at the corresponding points in time can be obtained from Table 5.

**Table 5:** Relative humidity (RH) and temperature (T) steps from the start of the respective main tests drying phases. The values between the steps are linearly interpolated

Time	Phase 1			Phase 2 and 3		
	RH (%)	T (K)	T ($^{\circ }$C)	RH (%)	T (K)	T ($^{\circ }$C)
Drying phase begins	99	293	20	99	293	20
6 h after beginning	41	303	30	41	303	30
8 h after beginning	30	320	47	30	320	47
From 20 h	20	353	80	15	353	80

For the Woodexter experiments, the climate data could be obtained from the corresponding report (Grüll et al. 2010), while for the CHMC experiments, the temperature and RH values were derived from ÖNORM EN 927-5 (2007).

Furthermore, $k_{{c}_w}$ was calibrated for each experimental setup. For the simulations of the Woodexter and CHMC experiments, $k_{{c}_w}$ was calibrated to cause minimal deviation between the simulation and experimental results at the end of the wetting phase and the drying phase (where performed), respectively. Based on these calibrated coefficients, an equation to estimate $k_{{c}_w}$ was derived (see Eq. (7)), which allowed for a more time-efficient calibration for the pretests and the main tests. For the pretests, $k_{{c}_w}$ was calibrated to obtain minimal variation between simulation and experimental results during the critical drying phase (from day 119 to 124). In this period, the most significant mass reductions and, thus, the largest moisture-induced stresses and deepest cracks occur. For the main tests, $k_{{c}_w}$ was calibrated to replicate minimal MC differences measured closest to the absorption surface at the end of the wetting phases. The identified $k_{{c}_w}$ values are summarized in Table 6.

**Table 6:** $k_{{c}_w}$ values calibrated for the coatings of the pretests and main tests, as well as the coatings presented in Grüll et al. (2010, 2015)

Pretests	UN	SAk	WAc					
$k_{{c}_w}$ (m s$^{-1}$)	$200 \cdot 10^{-10}$	$0.1 \cdot 10^{-10}$	$2.0 \cdot 10^{-10}$					

Main tests	UN	OWAc20	OWAc40	OWAc60				
$k_{{c}_w}$ (m s$^{-1}$)	$40 \cdot 10^{-10}$	$30 \cdot 10^{-10}$	$20 \cdot 10^{-10}$	$6 \cdot 10^{-10}$				

Woodexter	UN	P20	P50	P80	R50	W50	W100	
$k_{{c}_w}$ (m s$^{-1}$)	$70 \cdot 10^{-10}$	$30 \cdot 10^{-10}$	$27 \cdot 10^{-10}$	$27 \cdot 10^{-10}$	$24 \cdot 10^{-10}$	$20 \cdot 10^{-10}$	$19 \cdot 10^{-10}$	

CHMC	UN	BLO	TAc	TAcAk	TAk	LOSO	OAcAk	OAk
$k_{{c}_w}$ (m s$^{-1}$)	$42\cdot 10^{-10}$	$17 \cdot 10^{-10}$	$6.2 \cdot 10^{-10}$	$5.7 \cdot 10^{-10}$	$2.4 \cdot 10^{-10}$	$3.4 \cdot 10^{-10}$	$1.7 \cdot 10^{-10}$	$1.4 \cdot 10^{-10}$

UN = untreated, SAk = solvent-based alkyd coating, WAc = water-based acrylic coating, OWAc = opaque water-based acrylic formulation, BLO = boiled linseed oil, LOSO = linseed oil-stand oil, OAk = opaque solvent-based alkyd, OAcAk = opaque acrylic-/alkyd hybrid, TAk = transparent solvent-based alkyd, TAc = transparent acrylic, TAcAk = transparent acrylic-/alkyd hybrid; the numbers define the target thickness of the coating layers in $\upmu \hbox {m}$, while the single letters specify the colors of waterborne acrylic dispersions: P = brown, R = red and W = white

## Results and discussion

Simulating free water transport in wood is an enormous challenge due to wood’s inhomogeneity and structure. The introduced model, validated by several examples from the literature, is able to consider all major mechanisms and uses a reliable set of input parameters that are used for all the simulations (see Table S3 and S4 in Online Resource 1). To reproduce the experimental results, a maximum of two parameters had to be calibrated, with $k_{{c}_w}$ as the most significant coefficient. For the pretests and the main tests, the RH had to be determined, while $k_{{c}_v}$ had to be adjusted for the experiments of the Woodexter project. With regard to the complexity of simulating water uptake, a satisfying agreement between simulation and experimental results was achieved by calibrating up to a maximum of two parameters, as presented below.

At first, the MC and moisture mass *m* were determined based on the simulation output (bound water $c_b$, water vapor $c_v$ and free water $c_w$ concentration) as follows (the water vapor concentration $c_v$ is negligibly small and, thus, not considered):

6 $$\begin{aligned} \begin{aligned} \textrm{MC}&= (c_b + c_w)/ \rho _d \\ \rho&= \rho _{d} \cdot \frac{1 + \textrm{MC}}{1+0,84 \cdot \rho _{d} \cdot \textrm{MC}} \\ m&= \rho \cdot V \end{aligned} \end{aligned}$$

with $\rho _d$ as the dry density of wood, $\rho$ as the density of wood according to Kollmann (1951), and *V* as the volume of the test specimen. Unknown dimensional changes for the determination of *V* are considered with the expansion coefficients for swelling and shrinkage of wood (longitudinal = 0.01, radial and tangential = 0.24), presented in ÖNORM B 1995-1-1 (2019).

### k$_{\mathrm {c_w}}$ estimation

**Fig. 6:**
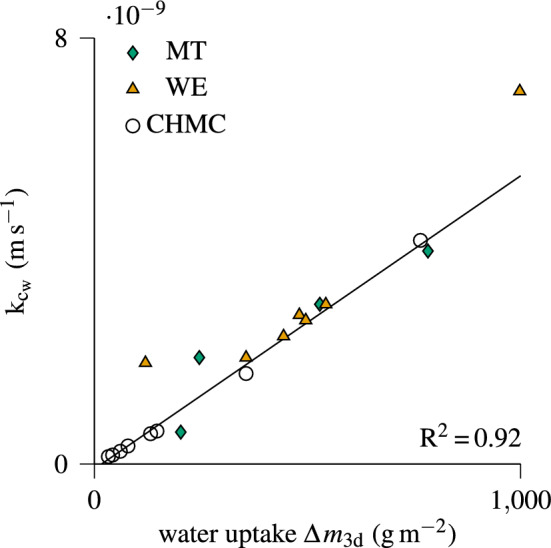
Comparison of the mass transfer coefficient of free water k$_{\mathrm {c_w}}$ and the water uptake after three days $\Delta \,m_{\textrm{3d}}$ for the simulated experiments (MT = main tests, WE = Woodexter, and CHMC = Comparison between Historical and Modern Coatings)

Simulating free water transport in case of infiltration with the model of Autengruber et al. (2020) requires a time-consuming calibration of the mass transfer coefficient of free water k$_{\mathrm {c_w}}$, which is the most significant parameter to replicate water uptake (Brandstätter et al. 2023). To reduce calibration time in future simulations, the Woodexeter and CHMC experiments were simulated (see Fig. S1 and S2 in Online Resource 1 for details), and used to obtain initial estimate values (see Table S1 and S2 in Online Resource 1). Analyzing the experimental and simulation results, as well as the literature, indicates that a comparison of given wood and coating properties should allow for an initial estimate. The most influencing factors include fiber orientation (Sedighi-Gilani et al. 2012; Siau 1984), wood type (Ahola et al. 1999; Gezici-Koç et al. 2017; Grüll et al. 2010; Sedighi-Gilani et al. 2012; Siau 1984; Virta et al. 2006), coating type (Ahola et al. 1999; de Meijer and Militz 2001; Ekstedt 2003; Grüll et al. 2013; Gezici-Koç et al. 2019) and their combinations (Angelski 2019), as well as number of coating layers (Hýsek et al. 2018; Angelski 2019; Grüll et al. 2010). Based on the highest similarity, one should be able to estimate k$_{\mathrm {c_w}}$. Detailed data about the coating configurations of the Woodexter and CHMC experiments are summarized in Table S1 and S2 (Online Resource 1) to simplify comparison. If additional information is given, the estimation of k$_{\mathrm {c_w}}$ can be improved, such as with the water uptake after three days. To illustrate the benefit, k$_{\mathrm {c_w}}$ is related to the water uptake after three days in Fig. 6. Examining the scatter plot in Fig. 6 indicates a relation between k$_{\mathrm {c_w}}$ and the water uptake after three days ($\Delta \,m_{\textrm{3d}}$), which can be described by the following linear equation:

7 $$\begin{aligned} \begin{aligned} \textrm{k}_{\mathrm {c_w}} = 5.51\!\cdot \!10^{-10} \Delta \,m_{\textrm{3d}} - 9.35\!\cdot \!10^{-11} \end{aligned} \end{aligned}$$

**Fig. 7:**
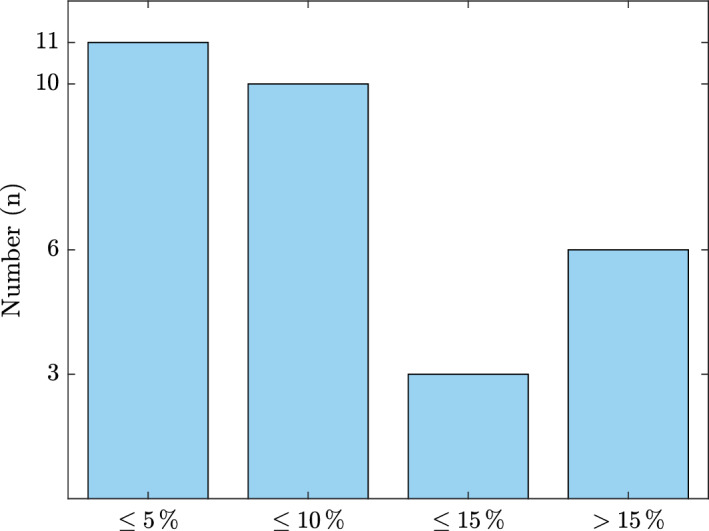
Histogram of the percentage deviation d according to Eq. (8) between the experimental and simulation water uptake

To evaluate the performance of Eq. (7), the experimental results of Ahola et al. (1999) and Angelski (2019) were simulated. Ahola et al. (1999) measured the water uptake of one uncoated and eleven coated specimens, while Angelski (2019) determined the amount of absorbed moisture for 18 boards with various coating configurations. Due to missing information, including the $s_\textrm{d}$ value, these experiments were simulated after those of Woodexter and CHMC. To determine an appropriate value, a parameter study was performed, revealing minimal influence of the $s_\textrm{d}$ value on water uptake in case of pure infiltration (no desorption during the process) and a minimum amount of absorbed moisture. Therefore, the $s_\textrm{d}$ value was assumed for the simulations of Ahola et al. (1999) and Angelski (2019) with 0.89 m (level from a reference coating system of the Woodexter project). Based on the additional simulation results, the deviation d between the experimental and the simulation water uptake was determined as follows:

8 $$\begin{aligned} \begin{aligned} \textrm{d} = \frac{ |\Delta \,m_{\textrm{3d,SIM}} - \Delta \,m_{\textrm{3d,EXP}} |}{\Delta \,m_{\textrm{3d,EXP}}} \cdot 100 \end{aligned} \end{aligned}$$

with $\Delta \,m_{\textrm{3d,SIM}}$ as the simulated and $\Delta \,m_{\textrm{3d,EXP}}$ as the experimental water uptake after three days. Figure 7 shows an overview of the result illustrated as a histogram, where 6 of 30 simulations show a significant deviation (“$>\,15\,\%$”). These 6 were coated specimens and characterized by minimal moisture uptake ($\Delta \textrm{m}_{\textrm{3d}} \le 34\mathrm {g\,m^{-2}}$). In these cases, it can be deduced that the $s_\textrm{d}$ value significantly influences the water uptake and, therefore, has to be known to sufficiently estimate the amount of absorbed moisture. The minimum amount of absorbed water, where $s_\textrm{d}$ did not influence the water uptake, was 112 g m$^{-2}$. Furthermore, Ahola et al. (1999) did not measure the dry density, which was assumed with 420 kg m$^{-3}$. Increasing this value decreased the deviation considerably, and consequently, an even better performance of the equation is expected. In addition, the performance of Eq. (7) was determined for a wetting phase lasting longer than three days to evaluate the influence of absorption time. Ahola et al. (1999) also measured the water uptake after seven days. Comparing the experimental and simulation results reveals an average overestimation of about 32 % (maximum: 47 %, minimum: 14 %). Therefore, the estimated k$_{\mathrm {c_w}}$ can be reduced for a longer simulation duration.

In addition, Eq. (7) was used to estimate k$_{\mathrm {c_w}}$ for the pre- and the main tests. For the main tests, the resulting values only led to a small deviation between the simulation and experimental results, allowing for a short calibration time (see Fig. 6). However, the first wetting phase of the pretests lasted only one day in contrast to the other experiments, and wetting phases that occurred later and lasted at least three days were not suitable as prior moisture changes have already biased the uptake. Therefore, significant differences between simulation and experimental results were observed, leading to a longer calibration time of k$_{\mathrm {c_w}}$ for the pretest’s results, which are presented in the following.

### Pretests

**Fig. 8:**
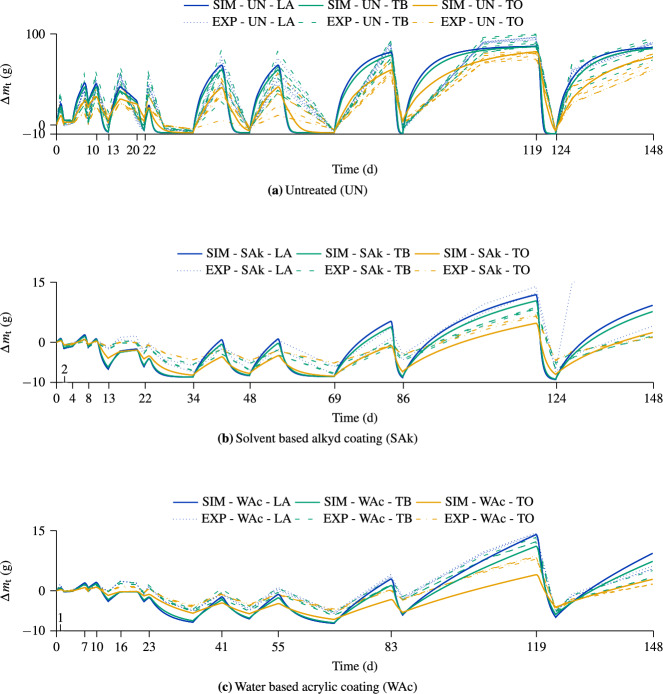
Comparison of the simulation (SIM) and experimental (EXP) moisture mass differences $\Delta \,m_\textrm{t}$ (= *m*(t) − *m*(t=0)) over time. The second and third terms of the legend entry define the coating type and configuration, respectively (UN = untreated, SAk = solvent-based alkyd coating, and WAc = water-based acrylic coating; LA = lateral area, TB = top and bottom surfaces, TO = only the top surface). The specified surfaces define the moisture absorption areas. Compare with Fig. 2 to see which boundary conditions are applied at which periods

Figure 8 shows the simulation and experimental results of the pretests. Since the mass variations between the boards can be significant (e.g., maximum initial difference = 54 g), the difference in mass over time $\Delta \,m_\textrm{t}$ (= *m*(t) − *m*(t=0)) is shown to improve comparability. For the pretests, an average dry density of 465 kg m$^{-3}$ with an assumed initial MC of 12 % was determined, based on the initial masses of the test specimens. Due to the significant variations in mass, the median was used instead of the mean value. As expected, for all coating configurations, the greater the proportion of unsealed surfaces, the greater the increase and decrease in $\Delta \,m_\textrm{t}$. However, comparing the LA and TB configuration results reveals marginal variations in $\Delta \,m_\textrm{t}$, showing minor contribution of the small side surfaces in moisture uptake and dry-out for both simulation and experiment.

Examining the experimental and simulation results for the untreated test specimens (UN configuration) at the end of the last two wetting cycles (see Fig. 8a), reveals $\Delta \,m_\textrm{t}$ not converging to the same limit, with a larger value for the experiments (except the UN-TO configuration). We assume that this is related to unknown exact dimensional changes of the test specimens. In addition, an error might be introduced by $\rho _d$, which is only estimated based on the initial weight rather than measured. Furthermore, the adsorption isotherm chosen for the simulations might not be ideal, which could result in a larger $c_b$ and, thus, in more moisture mass. However, despite the differences in the final value, the trend of the curves is similar, and the simulation results capture the water uptake of the experimental results.

For the simulation results of the coated test specimen (SAk and WAc), one can observe lower $\Delta \,m_\textrm{t}$ values compared to the experimental ones. On the one hand, this is caused by the RH, which is only assumed based on a temperature-dependent adsorption isotherm. On the other hand, the stepwise linear reduction of the RH and increase in temperature is solely adopted by former experiments conducted with a different drying oven since measurements are missing. Thus, the resulting reductions in $\Delta \,m_\textrm{t}$ are assumed to be overestimated. However, the determined k$_{\mathrm {c_w}}$ results in a sufficient change in $\Delta \,m_\textrm{t}$ that replicates the increase measured during the wetting phases adequately.

For each of the SAk-LA and SAk-TO configurations (see Fig. 8b), one experimental result is assumed to be an outlier due to too intense moisture uptake and, therefore, the $\Delta \,m_\textrm{t}$ development of only two boards is shown in each case. In addition, between 124 d and 148 d, an increased experimental moisture uptake of the SAk-LA configuration can be noticed, which was caused by damage in the sealing. Therefore, only a part of the increase is shown. Overall, the simulation results exhibit a significant correlation with the experimental ones.

In addition, stress simulations were performed to compare experimental and simulation crack development results. To minimize computation time, only the points in time with the maximum crack-prone volume were simulated, all occurring between days 119 and 124. It is important to note that several simplifications were made for the fracture simulations, including clear wood and homogeneous material properties, as well as an assumed crack development at an MC of 12 % for the multisurface failure criterion. In addition, the moisture field inducing the stresses remains unaffected by cracking. Therefore, the newly developed cracked surfaces are not in contact with the surrounding climate, which would influence the moisture field and, thus, the stresses. Furthermore, only linear elastic material behavior was considered. Implementing non-linear material behavior, including mechanosorption, viscoelasticity and plasticity, would reduce the induced stresses and, thus, the crack depth (Pech et al. 2024). Consequently, differences between the simulation and experimental results can be expected. For the experimental results, 10 out of 18 uncoated test specimens cracked, while no crack initiation was observed for the coated ones. Similar results were found for the simulations: while the coated models remained undamaged, cracks occurred in the uncoated ones. However, the cracks were up to 10 mm deep, whereas the cracks of the uncoated specimen appeared only superficial (the actual depth was not measured). It is assumed that another reason leads to a difference in experimental and simulation results. During the drying phase, the decrease rate in $\Delta \,m_\textrm{t}$ for the simulations is more significant compared to the experimental ones at the critical point in time. Observing the mass reduction between 10 d and 13 d as well as 20 d and 22 d reveals a considerably higher difference in $\Delta \,m_\textrm{t}$ for the simulation results. Therefore, larger moisture-induced stresses and, thus, deeper cracks occur.

Besides, inhomogeneities of wood could be considered to improve the agreement between simulation and experimental results. For example, knots could be considered in combination with a so-called phase field model, as presented in Pech et al. (2022a, 2022b). The model introduced allows the simulation of complex fracture phenomena for anisotropic materials and is largely independent of the mesh used, offering a promising alternative to methods based on a priori known crack paths.

Additional simulations of boards and GLT cross sections with the same climatic load and coating properties, intended for the main tests, revealed higher stresses for the boards. Therefore, to induce cracks in coated GLT test specimens during the experiments, a coating with a lower $s_\textrm{d}$ value had to be used. However, since no crack development could be observed for the coated boards, the $s_\textrm{d}$ value had to be even lower and the drying load adapted. Therefore, the oven temperature was increased to 80 $^{\circ }$C.

### Main tests

**Fig. 9:**
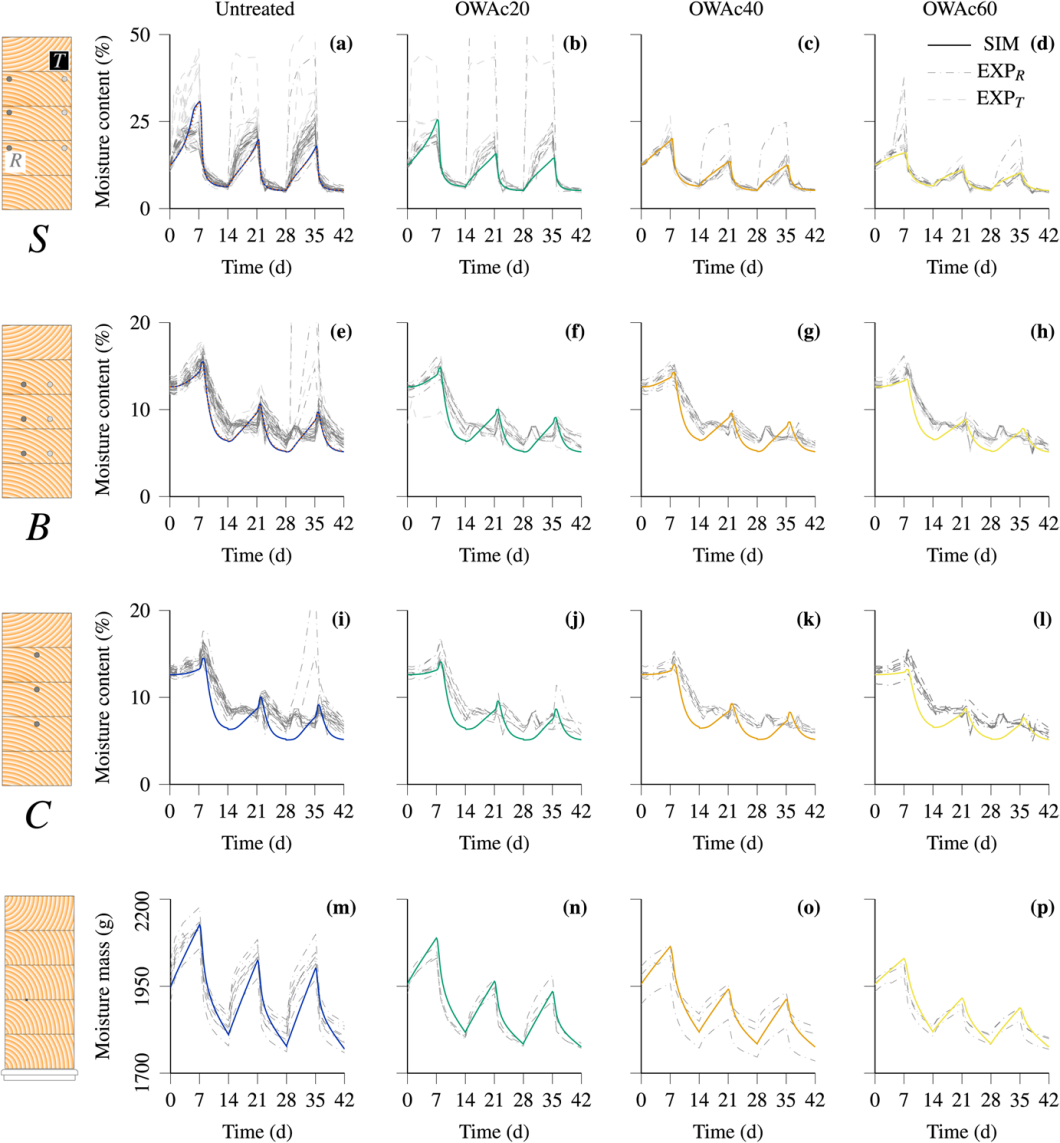
Simulation (SIM) and experimental (EXP) results, with colors defining the corresponding coating configuration. Coating: opaque white acrylic water-based experimental formulation (OWAc), with the numbers at the end defining the target thickness of the coating layers ($\upmu \hbox {m}$). The images on the left side help to illustrate to which measurement (point) the values refer to. *R* defines the measurement points closer to the predominantly radial surface (dark grey and dash-dotted lines), whereas *T* includes all points closer to the predominantly tangential surface (light grey and dashed curves). To show simulation results evaluated at the *R* layer, a dotted line is added in (**a**) and (**e**) as an example (color figure online)

Figure 9 presents the experimental and simulation results of the main tests, clustered by coating configuration and measurement point position. As depicted in Fig. 3, the MC was measured at three different depths (8 mm, 24 mm and 38.5 mm) from both side surfaces. Despite different fiber orientations, sufficiently similar simulation results are expected due to equal surface distances. Therefore, the results are clustered by depth, as illustrated by the cross sections on the left side in Fig. 9. The depth layers are labeled as *S*, *B* and *C* (Surface, Between and Center) and indexed with *R* and *T* to denote whether the measurement points are closer to the predominantly radial surface or the predominantly tangential surface. To improve perceptibility, the MC development of outliers is only partly shown. For the main tests, an average dry density of 470 kg m$^{-3}$ was calculated, and an initial MC of 12 % was assumed.

Before the simulation results are studied in detail, the RH, which is based on the experimental MC at the end of the drying phase and the desorption isotherm (see Eq. (1)), is examined. For the first drying phase, the RH was increased from 15 % to 20 % due to the following reasons. First, the MC at the end of the first wetting phase is considerably higher compared to the second and third. Therefore, it can be assumed that more water can evaporate from the test specimen in the following drying phase, leading to a higher average RH in this period compared to the other drying phases. Second, the measured average MC at the end of the first drying phase at the *S* layer is about 6.3 %, which corresponds to an RH of about 20 % considering the desorption isotherm. Thus, the stresses induced during the drying phase are less overestimated due to a higher MC, and the simulated crack development should deviate less from the experimental results.

Comparing the experimental and simulation results at the *S* layer (see Fig. 9a and b) reveals differences in the moisture uptake during the wetting phases. While the simulation MC development can be described by convex growth, the experimental water ingress is characterized by concave increase. In addition, the maximum MC at the end of the wetting phases is larger for the experiments. If wood is immersed into water, immense moisture uptake can be observed in the infiltration area due to capillary pressure, examined in several works (e.g., (Krabbenhøft and Damkilde 2004a; Sandberg and Salin 2012; Perré et al. 2022)). The model of Autengruber et al. (2020) used for the simulations is not able to consider this effect adequately. Therefore, the experimental and simulation MC can vary at the end of the wetting phases, especially close to the surface. This can be particularly observed for the untreated (see Fig. 9a) and the OWAc20 configuration (see Fig. 9b. However, the trend of the simulation water uptake above the FSP aligns well with the experimental results, and predominately convex growth can also be observed for the simulation results in Fig. 9c and d.

In the works of Virta et al. (2006), Niklewski et al. (2016) and Niklewski and Fredriksson (2021), the moisture development in boards exposed to rain was successfully simulated using a single-Fickian numerical approach. Notably, in Virta et al. (2006), the alignment between experimental and simulation results for the moisture profile close to the infiltration surface was good and, in certain cases, even excellent. When comparing the model used in this work and that of Virta et al. (2006), differences in the diffusion and permeability coefficients can be noticed. Thus, additional experiments to investigate the processes occurring in this region and thereby draw conclusions to adapt these coefficients could improve the performance of the model.

In addition, the MC measured at $S_R$ differs from $S_T$ due to the influence of the fiber orientation on moisture infiltration. However, this difference is less pronounced for the simulations (see dotted line in Fig. 9a and e). For the simulations, orientation-dependent moisture transport is considered, but deviations remain. A possible approach for an improved alignment would be a more precise implementation of the water ingress effect close to the absorption surface, considering the influence of the fiber orientation correspondingly.

For the simulation results at *B* and *C* (see Fig. 9e–l), changes in the moisture development occur at certain points in time. Each time the wetting phase ends, a significant MC increase can be noticed, although drying was initiated. This is related to the distance to the surfaces affected by the boundary conditions. A certain amount of time is required to reduce the MC in the corresponding depth. Until this occurs, the moisture accumulated at the surface can continue to diffuse into the cross section. However, the intensity of the increase in MC is related to the change in temperature. The higher the temperature, the quicker moisture diffuses through the cross section. It can be seen that the simulation results correctly reproduce the experimental ones.

Investigating the simulation MC in Fig. 9a at the end of the first wetting phase reveals a decrease in moisture uptake. This change in moisture absorption is caused by the FSP, which limits $c_b$. From this point on, only $c_w$ increases and, thus, the uptake is reduced. Similar behavior is noticed for simulation and experimental results. All in all, the simulation results show good agreement with the experimental ones.

**Fig. 10:**
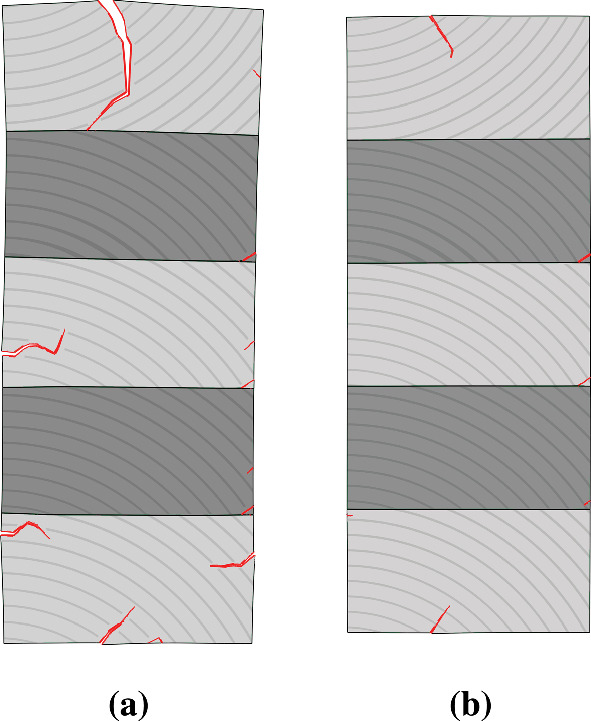
Result of the stress simulation at the critical point in time, exemplified for the uncoated configuration (**a**), and a coated one with an $s_\textrm{d}$ value of 0.08 m (**b**)

In addition to the moisture simulations, subsequent stress simulations, including XFEM and surface-based cohesive behavior, were performed to compare the experimental and simulation results in crack development (see Fig. 10). As for the pretest, primarily similarities in cracking should be expected, such as cracks initiating along the glue lines. For the simulations, cracks also occurred in the elements next to the glue lines, without cohesive surface failure, comparable to the experimental results. Thus, for both cases, failure of wood can be observed. Furthermore, similarities in deformation can be observed between the simulation results and the wooden test specimen. Comparing the simulation results with literature also shows agreement. Sandberg (1999) investigated the crack development of spruce test specimens on tangential and radial surfaces, which were subjected to an outdoor climate for 33 months. He discovered a greater number of cracks on the tangential surfaces compared to the radial ones, as can be seen in the simulation results.

## Conclusion and outlook

This work examined the moisture development below and above the fiber saturation point in (un)coated boards and glued laminated timber (GLT) members, as well as the resulting crack development, using both experiments and simulations. During the experiments, uncoated and coated Norway spruce boards (for coating pre-evaluation) and GLT beams (main tests) were subjected to wetting (water infiltration) and drying cycles. Moisture transport below and above the fiber saturation point could be simulated by using the multi-Fickian model introduced by some of the authors. As the mass transfer coefficient of free water k$_{\mathrm {c_w}}$ has been identified as the most significant parameter for the simulation of free water transport in a previous study, efforts were made to develop approaches simplifying the calibration process. For this purpose, additional experiments previously performed by some of the authors were examined and simulated. Based on the findings, k$_{\mathrm {c_w}}$ was more efficiently calibrated for the main tests. Subsequently, experimental and simulation results were compared.

In addition to the changes in moisture content (MC), the initiation and propagation of cracks caused by the intense drying load in the timber elements were compared to the simulation results. For this purpose, additional stress analyses, including linear elastic traction-separation behavior and XFEM (allowing for discrete cracking), were performed, using the results of the MC simulations as load. The strength of wood was defined by a multisurface failure criterion developed by some of the authors and moisture-dependent stiffness parameters determined in earlier studies were used. The main conclusions are summarized as follows:

- The simulation results show qualitatively valuable alignment with the experimental ones for both water absorption and dry-out in coated wood specimens. Hence, simulations are also suitable to examine moisture transport including free water in coated GLT members.
- For an initial estimation of k$_{\mathrm {c_w}}$ in case of coated wooden members, one has to consider several influencing factors, including wood type, fiber orientation, coating type and their combination, as well as number of coating layers. If the water uptake after three days is known, a more accurate prediction of k$_{\mathrm {c_w}}$ is possible, allowing for a reasonable simulation result in appropriate time.
- In case of solely simulating water infiltration in coated wood specimens, a range of $s_\textrm{d}$ values can be used, and still, a strong agreement between simulation and experimental results can be achieved, provided there is a minimum water uptake. Therefore, less information for simulations may be sufficient.

The results near the infiltration surface showed immense water absorption and a significant influence of the fiber orientation on moisture distributions, with the largest deviations between simulation and experimental results being observed in this area. To enhance the accuracy of future simulations, the processes occurring in this region should be analyzed more thoroughly. This investigation could lead to the derivation of more precise diffusion and permeability parameters or functions, as well as mass transfer coefficients, ultimately improving the numerical model.

Moreover, more experiments investigating moisture uptake of coated wood specimens should be simulated to verify the quality of the k$_{\mathrm {c_w}}$ estimation function. Experimental results characterized by minimal moisture uptake could be simulated to determine the moisture mass threshold at which the influence of the $s_\textrm{d}$ value on water absorption becomes negligible.

## Supplementary Information

Below is the link to the electronic supplementary material.

Supplementary file 1 (pdf 401 KB)

## Data Availability

On request.

## List of symbols

$\Delta \,m_\textrm{t}$: Difference in moisture mass over time (= *m*(t) − *m*(t = 0))(g)
$\Delta \,m_{\textrm{3d,EXP}}$: Water uptake after three days from experimental results
$\Delta \,m_{\textrm{3d,SIM}}$: Water uptake after three days from simulation results
$\Delta \,m_{\textrm{3d}}$: Water uptake after three days (= *m*(t = 3) − *m*(t = 0))(g)
$\phi _v$: Boundary flux of water vapor ($\mathrm {kg\,m^{-2}\,s^{-1}}$)
$\phi _w$: Boundary flux of free water ($\mathrm {kg\,m^{-2}\,s^{-1}}$)
$\rho _d$: Density of dry wood ($\mathrm {kg\,m^{-3}}$)
$\rho$: Density of wood ($\mathrm {kg\,m^{-3}}$)
$c_b$: Bound water concentration ($\mathrm {kg\,m^{-3}}$)
$c_v$: Water vapor concentration ($\mathrm {kg\,m^{-3}}$)
$c_w$: Free water concentration ($\mathrm {kg\,m^{-3}}$)
$c_{v,0}$: Surrounding water vapor concentration ($\mathrm {kg\,m^{-3}}$)
$c_{w,0}$: Surrounding free water concentration ($\mathrm {kg\,m^{-3}}$)
$D_{air}$: Diffusion coefficient of water vapor (m$^{2}$ s$^{-1}$)
$f_{i,s}$: Isotherm shape factors (−)
$f_{lum}$: Volume fraction of the lumen (−)
*V*: Volume (m$$^3$$)
$k_{c_v}$: Mass transfer coefficient for water vapor ($\mathrm {m\,s^{-1}}$)
$k_{c_w}$: Mass transfer coefficient for free water ($\mathrm {m\,s^{-1}}$)
$k_{{c}_v,coat}$: Considers the influence of potential coatings for $k_{c_v}$ ($\mathrm {m\,s^{-1}}$)
$k_{{c}_v,surf}$: Considers convection and airflow depending on airspeed for $k_{c_v}$ ($\mathrm {m\,s^{-1}}$)
*m*: Moisture mass (g)
$s_d$: Diffusion equivalent air layer thickness (m)
*Sh*: Sherwood number (−)
T: Temperature (K)
*L*: Characteristic length (m)
BLO: Boiled linseed oil
CHMC: Experimental series: “comparison of historic and modern coatings”
d: Deviation between the experimental and the simulation water uptake after three days
EMC: Equilibrium moisture content (%)
FSP: Fiber saturation point (%)
LOSO: Linseed oil-stand oil
MC: Moisture content (%)
OAcAk: Opaque acrylic-/alkyd hybrid
OAk: Opaque solvent-based alkyd
OWAc: Opaque white acrylic water-based experimental formulation
RH: Relative humidity (%)
SAk: Solvent-based alkyd stain
TAc: Transparent acrylic
TAcAk: Transparent acrylic-/alkyd hybrid
TAk: Transparent solvent-based alkyd
UN: Untreated
WAc: Water-based acrylic paint
XFEM: Extended finite element method
