# Supplementary material for: Investigation of cyclic water infiltration and dry-out in coated spruce using finite-element simulations
Source: Wood Sci Technol. 2025 Jan 22;59(1):25. doi: 10.1007/s00226-025-01629-7 (PMC11754350; doi:10.1007/s00226-025-01629-7)
Supplement: Supplementary file 1 — (pdf 401 KB) [file 226_2025_1629_MOESM1_ESM.pdf]

# Investigation of cyclic water infiltration and dry-out in coated spruce using finite-element simulations (supplematry material)

Florian Brandstätter[1*], Magdalena Senoner[2], Markus Lukacevic[1], Maximilian Autengruber[1], Michael Truskaller[2], Gerhard Grüll[2] and Josef Füssl[1]

[1*]TU Wien, Institute for Mechanics of Materials and Structures, Karlsplatz 13, Vienna, 1040, Austria.
[2*]Holzforschung Austria, Franz Grill-Strasse 7, Vienna, 1030, Austria.

*Corresponding author E-mail: florian.brandstaetter@tuwien.ac.at;
E-mail: m.senoner@holzforschung.at; markus.lukacevic@tuwien.ac.at; maximilian.autengruber@tuwien.ac.at; m.truskaller@holzforschung.at; g.gruell@holzforschung.at; josef.fuessl@tuwien.ac.at;

# Woodexter - additional information

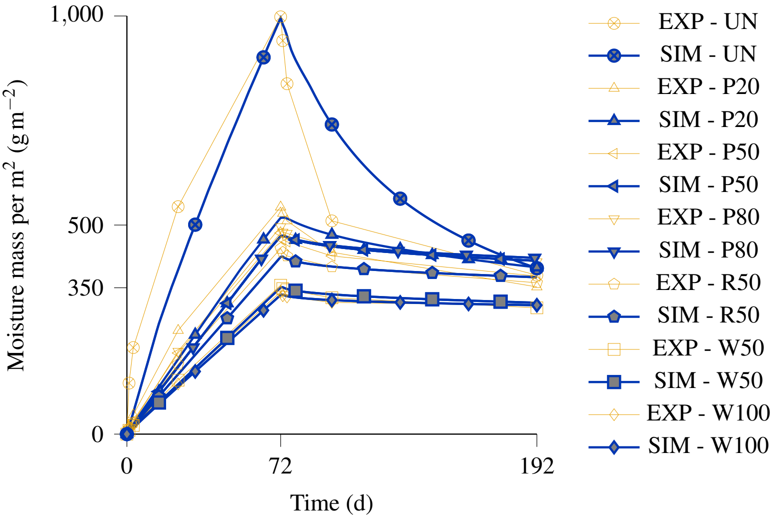


**Fig. S1**: Development of the moisture mass development per $\mathrm{m}^2$ for the simulation and experimental results

**Table S1**: Data overview of coatings studied during the Woodexter experiments (Grüll et al, 2010). The numbers define the target thickness of the coating layers in $\mu$m, while the single letters specify the colors of waterborne acrylic dispersions: P = brown, R = red and W = white.

| coatings | $k_{c_w}$ ($\mathrm{m\,s^{-1}}$) | $s_\mathrm{d}$ (m) | permeance $W$ ($\mathrm{kg\,m^{-2}\,s^{-1}\,Pa^{-1}}$) | description |
|---|---|---|---|---|
| P20 | $30 \cdot 10^{-10}$ | 0.24 | $8.2 \cdot 10^{-10}$ | one-layered, water-based acrylic stain, semi-transparent |
| P50 | $28 \cdot 10^{-10}$ | 0.69 | $2.9 \cdot 10^{-10}$ | two-layered, water-based acrylic stain, semi-transparent |
| P80 | $27 \cdot 10^{-10}$ | 0.85 | $2.4 \cdot 10^{-10}$ | two-layered, water-based acrylic stain, semi-transparent |
| R50 | $24 \cdot 10^{-10}$ | 0.86 | $2.3 \cdot 10^{-10}$ | two-layered, water-based acrylic paint, opaque, red |
| W50 | $20 \cdot 10^{-10}$ | 0.89 | $2.2 \cdot 10^{-10}$ | two-layered, water-based acrylic paint, opaque, white |
| W100 | $19 \cdot 10^{-10}$ | 1.62 | $1.2 \cdot 10^{-10}$ | two-layered, water-based acrylic paint, opaque, white |

According to EN ISO 12572 (2016), the permeance $W$ is defined as follows:

$$W = \frac{D_{air}}{s_\mathrm{d} R_D T} \tag{1}$$

$$D_{air} = \left(2.31 \cdot 10^{-5} \frac{p_{atm}}{p_{atm} + p_{v_{air}}} \left(\frac{T}{273}\right)^{1.81}\right) \tag{2}$$

with $R_D$ as the gas constant of water vapour ($\mathrm{Pa\,kg^{-1}\,K^{-1}}$) and $D_{air}$ as the diffusion coefficient of water vapor ($\mathrm{m^2\,s^{-1}}$, Schirmer, 1938).

## CHMC - additional information

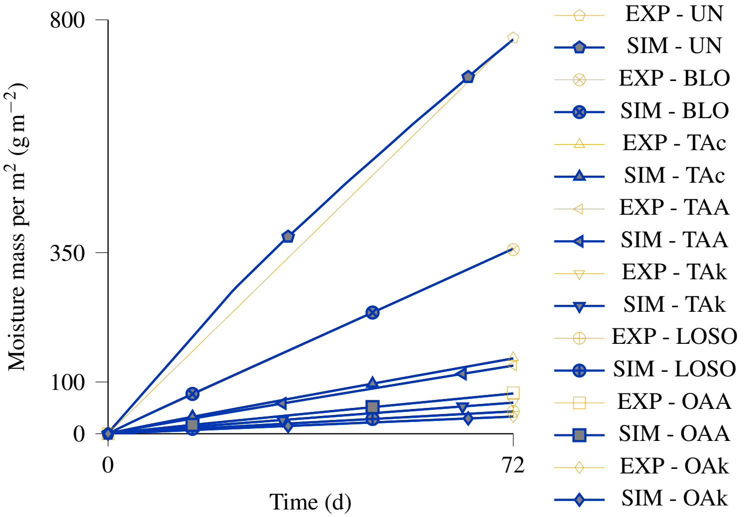


**Fig. S2**: Development of the moisture mass development per m$^2$ for the simulation and experimental results

**Table S2**: Data overview of coatings examined during the CHMC experiments (Grüll et al, 2015) (BLO = boiled linseed oil, LOSO = linseed oil-stand oil, OAk = opaque solvent-based alkyd, OAcAk = opaque acrylic-/alkyd hybrid, TAk = transparent solvent-based alkyd, TAc = transparent acrylic, TAcAk = transparent acrylic-/alkyd hybrid).

| coatings | $k_{c_w}$ ($\mathrm{m\,s^{-1}}$) | $s_\mathrm{d}$ (m) | permeance ($\mathrm{kg\,m^{-2}\,s^{-1}\,Pa^{-1}}$) | description |
|---|---|---|---|---|
| BLO | $17 \cdot 10^{-10}$ | 0.7 | $2.8 \cdot 10^{-10}$ | three layers, boiled linseed oil, transparent |
| TAc | $6.2 \cdot 10^{-10}$ | 0.9 | $2.2 \cdot 10^{-10}$ | three layers, transparent acrylic resin |
| TAcAk | $5.7 \cdot 10^{-10}$ | 1.3 | $1.5 \cdot 10^{-10}$ | three layers, transparent acrylic-/alkyd resin hybrid |
| TAk | $2.4 \cdot 10^{-10}$ | 2.5 | $0.8 \cdot 10^{-10}$ | three layers, transparent solvent-based alkyd resin |
| LOSO | $3.4 \cdot 10^{-10}$ | 3.4 | $0.6 \cdot 10^{-10}$ | three layers, linseed oil-stand oil, opaque |
| OAcAk | $1.7 \cdot 10^{-10}$ | 3.4 | $0.6 \cdot 10^{-10}$ | three layers, opaque acrylic-/alkyd resin hybrid |
| OAk | $1.4 \cdot 10^{-10}$ | 6.9 | $0.3 \cdot 10^{-10}$ | three layers, opaque solvent based alkyd resin |

# Material properties and constitutive equations for the multi-Fickian model

To calculate the thermodynamic properties for specific isobaric heat capacities $c_p$ and enthalpies $h$ the reference state for the constitutive equations is set to 273 K and 101 325 Pa, respectively.

**Table S3**: Material parameters as well as constitutive equations for determination of the moisture and heat transport.

| Property | Value/Constitutive equation | Ref. |
|---|---|---|
| Moisture content | $\mathrm{MC} = \frac{c_b}{\rho_d}$ | |
| Bound water flux | $\boldsymbol{J_b} = -\boldsymbol{D_b} \cdot \frac{\partial c_b}{\partial \boldsymbol{x}} - \boldsymbol{D_{bT}} \cdot \frac{\partial T}{\partial \boldsymbol{x}}$ | (Frandsen, 2007) |
| Bound water diffusion tensor | $\boldsymbol{D_b} = \boldsymbol{D_0} \exp\left(\frac{-E_b}{R\,T}\right)$ | (Frandsen, 2007) |
| Bound water diffusion tensor (Soret effect) | $\boldsymbol{D_{bT}} = \boldsymbol{D_0} \frac{c_b\,E_b}{R\,T^2} \exp\left(\frac{-E_b}{R\,T}\right)$ | (Frandsen, 2007) |
| Activation energy of bound water | $E_b = 38500 - 29000\,\mathrm{MC}$ | (Siau, 1984) |
| Universal gas constant | $R = 8.314\,\mathrm{J\,mol^{-1}\,K^{-1}}$ | |
| Water vapor flux | $\boldsymbol{J_v} = -\boldsymbol{D_v} \cdot \frac{\partial c_v}{\partial \boldsymbol{x}}$ | |
| Water vapor diffusion tensor | $\boldsymbol{D_v} = \boldsymbol{\xi} \left(2.31 \cdot 10^{-5} \frac{p_{atm}}{p_{atm} + p_{v_{air}}} \left(\frac{T}{273}\right)^{1.81}\right)$ | (Schirmer, 1938; Frandsen et al, 2007; Krabbenhøft and Damkilde, 2004) |
| Water vapor pressure | $p_{v_{air}} = c_v \frac{R\,T}{M_{H_2O}}$ | |
| Atmospheric air pressure | $p_{atm} = 101\,325\,\mathrm{Pa}$ | |
| Molar mass of water | $M_{H_2O} = 18.015\,\mathrm{g\,mol^{-1}}$ | |
| Moist density of wood | $\rho = \rho_d \frac{1+u}{1+0.84\,c_b}$ | (Kollmann, 1951) |
| Volume proportion of the cell lumen | $f_{lum} = 1 - \frac{\rho_{moist}}{\rho_{cwm}}$ | |
| Density of the pure cell wall material | $\rho_{cwm} = 1530\,\mathrm{kg\,m^{-3}}$ | (Siau, 1984; Eitelberger et al, 2011) |
| Free water flux | $\boldsymbol{J_w} = -\rho_{H_2O} \frac{\boldsymbol{K_r} \cdot \boldsymbol{K_w}}{\mu_w} \cdot \frac{\partial P_g - P_c}{\partial \boldsymbol{x}}$ | |
| Density of water | $\rho_{H_2O} = 1000\,\mathrm{kg\,m^{-3}}$ | |
| Dynamic viscosity of water | $\mu_w = 2.414 \cdot 10^{-5} \cdot 10^{\frac{247.8}{T-140}}$ | (Touloukian et al, 1975) |
| Capillary pressure | $P_c = 12400\,S_w^{-0.61}.$ | (Spolek and Plumb, 1981) |
| Heat flux | $\boldsymbol{f} = -\boldsymbol{K} \cdot \frac{\partial T}{\partial \boldsymbol{x}}$ | |
| Conduction tensor | $\boldsymbol{K} = \boldsymbol{K_0}\,(0.142 + 0.46\,\mathrm{MC})$ | (Perré and Turner, 1999) |
| Heat capacity of the cell wall material | $c_{p_s} = -0.60453 + 0.006714\,T$ | (Yang, 2000) |
| Enthalpy of water vapor | $h_v = 2060.5 + 1.3798\,T + 0.84808 \cdot 10^{-4}\,T^2$ | (Eitelberger, 2011) |
| Specific enthalpy of bound water | $h_b = 4.185\,(T - 273\,\mathrm{K}) - 1146.4\,\exp(-14.48\,\mathrm{MC})$ | (Skaar, 1988; Eitelberger, 2011) |
| Average enthalpy of bound water | $\overline{h}_b = -1143.1 + 4.185\,T - \frac{79.172\,\rho_d\,(1-\exp(-14.48\,\mathrm{MC}))}{c_b}$ | (Stanish et al, 1986; Turner, 1996; Eitelberger, 2011) |

**Table S4**: Diagonal components of the material parameter tensors

| Parameter | Component | | | Ref. |
|---|---|---|---|---|
| | L | R | T | |
| $\boldsymbol{K_w}$ | $1 \cdot 10^{-12}$ | $2 \cdot 10^{-15}$ | $1 \cdot 10^{-15}$ | (Perré and Turner, 1999) |
| $\boldsymbol{K_r}$ | $S_w^8$ | $S_w^3$ | $S_w^3$ | (Perre et al, 1993) |
| $\boldsymbol{D_0}$ | $2.5 \cdot 7 \cdot 10^{-6}$ | $7 \cdot 10^{-6}$ | $7 \cdot 10^{-6}$ | (Fortino et al, 2013) |
| $\boldsymbol{\xi}$ | 0.9 | 0.11 | 0.11 | (Dvinskikh et al, 2011) |
| $\boldsymbol{K_0}$ | 2 | 1 | 1 | (Perre et al, 1993) |

# Elasticity tensor of spruce

**Table S5**: Tensor components $C_{iiii}$ [MPa] for spruce elasticity at 293 K with a dry density of 420 kg m$^{-3}$ (Hofstetter et al, 2005).

| MC [%] | $C_{LLLL}$ | $C_{RRRR}$ | $C_{TTTT}$ | $C_{LLRR}$ | $C_{RRTT}$ | $C_{TTLL}$ | $C_{LRLR}$ | $C_{LTLT}$ | $C_{RTRT}$ |
|---|---|---|---|---|---|---|---|---|---|
| 3 | 13981.39 | 1127.15 | 755.30 | 343.76 | 272.01 | 520.26 | 396.68 | 389.40 | 53.70 |
| 4 | 13824.31 | 1094.04 | 733.20 | 337.33 | 266.94 | 504.70 | 385.64 | 378.57 | 52.23 |
| 5 | 13669.80 | 1061.40 | 711.42 | 330.88 | 261.87 | 489.33 | 374.67 | 367.80 | 50.79 |
| 6 | 13517.80 | 1029.22 | 689.96 | 324.41 | 256.78 | 474.16 | 363.78 | 357.11 | 49.37 |
| 7 | 13368.23 | 997.51 | 668.82 | 317.92 | 251.67 | 459.21 | 352.97 | 346.49 | 47.97 |
| 8 | 13221.06 | 966.26 | 647.98 | 311.40 | 246.55 | 444.47 | 342.25 | 335.97 | 46.60 |
| 9 | 13076.22 | 935.48 | 627.46 | 304.86 | 241.40 | 429.95 | 331.63 | 325.55 | 45.24 |
| 10 | 12933.66 | 905.17 | 607.25 | 298.29 | 236.23 | 415.66 | 321.12 | 315.23 | 43.91 |
| 11 | 12793.34 | 875.33 | 587.34 | 291.68 | 231.03 | 401.60 | 310.72 | 305.02 | 42.59 |
| 12 | 12655.21 | 845.95 | 567.74 | 285.04 | 225.80 | 387.77 | 300.45 | 294.94 | 41.29 |
| 13 | 12519.23 | 817.05 | 548.45 | 278.37 | 220.55 | 374.19 | 290.31 | 284.99 | 40.00 |
| 14 | 12385.35 | 788.61 | 529.47 | 271.66 | 215.27 | 360.84 | 280.32 | 275.18 | 38.73 |
| 15 | 12253.54 | 760.65 | 510.79 | 264.92 | 209.95 | 347.74 | 270.47 | 265.51 | 37.47 |
| 16 | 12123.75 | 733.16 | 492.42 | 258.14 | 204.61 | 334.88 | 260.78 | 256.00 | 36.23 |
| 17 | 11995.96 | 706.15 | 474.35 | 251.33 | 199.24 | 322.26 | 251.25 | 246.64 | 35.01 |
| 18 | 11870.13 | 679.60 | 456.59 | 244.48 | 193.84 | 309.90 | 241.89 | 237.46 | 33.79 |
| 19 | 11746.22 | 653.53 | 439.14 | 237.61 | 188.41 | 297.77 | 232.71 | 228.44 | 32.60 |
| 20 | 11624.20 | 627.93 | 421.99 | 230.71 | 182.96 | 285.89 | 223.70 | 219.60 | 31.41 |
| 21 | 11504.05 | 602.80 | 405.15 | 223.78 | 177.49 | 274.26 | 214.88 | 210.94 | 30.24 |
| 22 | 11385.74 | 578.14 | 388.62 | 216.82 | 171.99 | 262.86 | 206.25 | 202.47 | 29.08 |
| 23 | 11269.23 | 553.95 | 372.39 | 209.85 | 166.48 | 251.71 | 197.81 | 194.18 | 27.94 |
| 24 | 11154.50 | 530.22 | 356.46 | 202.86 | 160.95 | 240.79 | 189.56 | 186.09 | 26.81 |
| 25 | 11041.52 | 506.95 | 340.83 | 195.86 | 155.41 | 230.11 | 181.51 | 178.18 | 25.70 |
| 26 | 10930.28 | 484.14 | 325.51 | 188.86 | 149.86 | 219.66 | 173.66 | 170.47 | 24.60 |
| 27 | 10820.74 | 461.80 | 310.49 | 181.85 | 144.30 | 209.44 | 166.00 | 162.96 | 23.52 |
| 28 | 10712.87 | 439.90 | 295.77 | 174.84 | 138.75 | 199.44 | 158.55 | 155.64 | 22.45 |
| 29 | 10606.66 | 418.46 | 281.35 | 167.84 | 133.20 | 189.67 | 151.29 | 148.52 | 21.40 |
| 30 | 10502.09 | 397.46 | 267.22 | 160.84 | 127.65 | 180.11 | 144.23 | 141.59 | 20.36 |

# Nomenclature

CHMC ....... experimental series: "comparison of historic and modern coatings" (Grüll et al, 2015)

# References

Dvinskikh SV, Henriksson M, Mendicino AL, et al (2011) Nmr imaging study and multi-fickian numerical simulation of moisture transfer in norway spruce samples. Engineering Structures 33:3079–3086. https://doi.org/https://doi.org/10.1016/j.engstruct.2011.04.011

Eitelberger J (2011) A multiscale material description for wood below the fiber saturation point with particular emphasis on wood-water interactions. PhD thesis, Vienna University of Technology

Eitelberger J, Hofstetter K, Dvinskikh S (2011) A multi-scale approach for simulation of transient moisture transport processes in wood below the fiber

saturation point. Composites Science and Technology 71(15):1727–1738. https://doi.org/https://doi.org/10.1016/j.compscitech.2011.08.004

EN ISO 12572 (2016) Hygrothermal performance of building materials and products – Determination of water vapour transmission properties – Cup method

Fortino S, Genoese A, Genoese A, et al (2013) Numerical modelling of the hygro-thermal response of timber bridges during their service life: A monitoring case-study. Construction and Building Materials 47:1225–1234. https://doi.org/https://doi.org/10.1016/j.conbuildmat.2013.06.009

Frandsen HL (2007) Selected constitutive models for simulating the hygromechanical response of wood. PhD thesis, Aalborg University

Frandsen HL, Damkilde L, Svensson S (2007) A revised multi-fickian moisture transport model to describe non-fickian effects in wood. Holzforschung 61:563–572. https://doi.org/doi:10.1515/HF.2007.085

Grüll G, Truskaller M, Podgorski L, et al (2010) WOODEXTER – Work Package 3: Interaction of wood and coatings – effect on the performance of wood products. Tech. rep., Holzforschung Austria

Grüll G, Fürhapper C, Aschacher G, et al (2015) Historische und moderne Beschichtungssysteme – Ein technischer und ökologischer Vergleich. Tech. rep., Holzforschung Austria, URL https://www.fcio.at/media/8838/hfa_wiener_holzschutztage_2015-historische_und_moderne_beschichtungssysteme.pdf

Hofstetter K, Hellmich C, Eberhardsteiner J (2005) Development and experimental validation of a continuum micromechanics model for the elasticity of wood. European Journal of Mechanics - A/Solids 24(6):1030–1053. https://doi.org/https://doi.org/10.1016/j.euromechsol.2005.05.006

Kollmann F (1951) Technologie des Holzes und der Holzwerkstoffe: 1. Band. Springer, Berlin, Heidelberg, URL https://books.google.at/books?id=T5GTBwAAQBAJ

Krabbenhøft K, Damkilde L (2004) A model for non-fickian moisture transfer in wood. Materials and Structures 37(9):615–622. URL http://dx.doi.org/10.1007/BF02483291

Perre P, Moser M, Martin M (1993) Advances in transport phenomena during convective drying with superheated steam and moist air. International Journal of Heat and Mass Transfer 36(11):2725–2746. https://doi.org/https://doi.org/10.1016/0017-9310(93)90093-L

Perré P, Turner IW (1999) A 3-d version of transpore: a comprehensive heat and mass transfer computational model for simulating the drying of porous media. International Journal of Heat and Mass Transfer 42(24):4501–4521. https://doi.org/https://doi.org/10.1016/S0017-9310(99)00098-8

Schirmer R (1938) Die Diffusionszahl von Wasserdampf-Luft-Gemischen und die Verdampfungsgeschwnidigkeit. VDI Beiheft Verfahrenstechnik 1938(6):170–177

Siau JF (1984) Transport Processes in Wood. Springer, Berlin, Heidelberg, https://doi.org/https://doi.org/10.1007/978-3-642-69213-0

Skaar C (1988) Wood-Water Relations. Springer-Verlag Berlin Heidelberg, https://doi.org/10.1007/978-3-642-73683-4

Spolek GA, Plumb OA (1981) Capillary pressure in softwoods. Wood Science and Technology 15(3):189–199. https://doi.org/https://doi.org/10.1007/BF00353471

Stanish MA, Schajer GS, Kayihan F (1986) A mathematical model of drying for hygroscopic porous media. AIChE J 32(8):1301–1311. https://doi.org/10.1002/aic.690320808

Touloukian Y, Saxena S, Hestermans P (1975) Thermophysical properties of matter-the tprc data series. volume 11. viscosity. Tech. rep., THERMOPHYSICAL AND ELECTRONIC PROPERTIES INFORMATION ANALYSIS CENTER ...

Turner IW (1996) A two-dimensional orthotropic model for simulating wood drying processes. Applied Mathematical Modelling 20(1):60–81. https://doi.org/https://doi.org/10.1016/0307-904X(95)00106-T

Yang QX (2000) Study on the specific heat of wood by statistical mechanics. Journal of Forestry Research 11(4):265–268. https://doi.org/https://doi.org/10.1007/BF02844975
